# Budget impact of expanded hemodialysis versus high-flux hemodialysis using real-world evidence in Colombia

**DOI:** 10.1186/s12962-026-00754-9

**Published:** 2026-05-02

**Authors:** Mauricio Sanabria, Jasmin Vesga, Angela Rivera, Peter Rutherford, Hoover Quitian, Bengt Lindholm

**Affiliations:** 1 Renal Care Services Latin-America, Carrera 7C bis N 141A-36, Bogota D.C, Colombia; 2 Renal Care Services Colombia, Bucaramanga, Colombia; 3 Vantive, Deerfield, Illinois USA; 4 Vantive, Zurich, Switzerland; 5 Faculty of Economics of the Universidad Nacional, Bogota D.C, Colombia; 6 Renal Medicine and Baxter Novum, Karolinska Institutet, Stockholm, Sweden

**Keywords:** Budget impact analysis, Hemodialysis, Expanded hemodialysis, End-stage kidney disease, Real-world evidence, Hospitalization

## Abstract

**Background:**

Expanded hemodialysis (HDx) using medium cut-off dialyzers has been associated with lower all-cause hospitalization rates compared to conventional high-flux hemodialysis. Reductions in hospitalization frequency represent a major driver of healthcare expenditures and may contribute to improved budget sustainability in resource-constrained healthcare systems. The objective of this study was to estimate the budget impact of adopting HDx from the perspective of the Colombian healthcare system, using real-world evidence.

**Methods:**

A budget impact analysis was developed according to the ISPOR guidelines using a tool built in Microsoft Excel. Clinical effectiveness inputs were derived from a multicenter cohort study (COREXH-E), including an extended dataset and a difference-in-differences analysis to estimate the effect of HDx on hospitalization rate while accounting for unobserved confounding. Cost inputs were obtained from national administrative databases, including hospitalization costs and bundled dialysis payments. The analysis adopted a one-year time horizon and a third-party payer perspective representing the Colombian healthcare system. Budget impact scenarios were evaluated, assuming HDx market uptake rates of 5%, 10%, 20%, and 50%. Deterministic and probabilistic sensitivity analyses were performed to assess parameter uncertainty.

**Results:**

Adoption of HDx was associated with net cost savings for the Colombian healthcare system across all uptake scenarios. Under hospitalization rates observed in the real-world cohort, estimated annual savings ranged from USD 472,756 to USD 4,727,564 as HDx uptake increased from 5% to 50%. In scenarios reflecting higher hospitalization rates observed in the general Colombian hemodialysis population, annual savings ranged from USD 1,344,401 to USD 13,444,010. Cost savings were primarily driven by reductions in hospitalization frequency. Probabilistic sensitivity analysis showed that HDx was cost-saving in 97.8% of simulations.

**Conclusions:**

This study suggests that expanded hemodialysis could result in short-term cost savings for the Colombian healthcare system by reducing hospitalization-related costs without increasing dialysis expenses.

**Supplementary Information:**

The online version contains supplementary material available at 10.1186/s12962-026-00754-9.

## Introduction

Chronic kidney disease with renal failure is a health condition that represents a significant challenge, requiring considerable medical and financial resources [1–2]. The high resource utilization per patient is driven not only by kidney replacement therapy (KRT) itself but also by the considerable morbidity and comorbidity burden observed in this population. In chronic hemodialysis programs, the current standard of care could be defined as ensuring three weekly hemodialysis sessions, lasting at least four hours, using water treated according to AAMI standards, and using high-flux hemodialyzers [3–4]. In addition, and due to the health condition of the patients, even if they are adequately treated, some complications require, on average, between 0.7 and 1.3 hospitalizations per patient per year, numbers that can increase if they do not receive timely and adequate medical care [5]. Consequently, hospitalizations constitute a major driver of healthcare expenditures in chronic hemodialysis programs.

Recent technological advances have introduced medium cut-off membranes (MCO), which, compared to high-flux membranes (HF), are more effective at removing larger middle molecules from the blood [6–9]. This advancement, known as Expanded Hemodialysis (HDx), ^10–11^ can be implemented without modifications to dialysis infrastructure or staffing, as it requires only replacement of the dialyzer membrane [10–13]. More broadly, advances in dialysis technologies, including the integration of tools such as artificial intelligence to support clinical decision-making, aim to improve the management of complications in this population [14].

Observational evidence has suggested that HDx may be associated with reductions in hospitalization rates and hospital days compared with high-flux hemodialys [8–12]. These clinical benefits are also associated with potential economic implications. A randomized clinical trial in the United States estimated annual cost reductions by USD 6,091 per patient, primarily due to fewer hospitalization events [15]. Similarly, a retrospective Colombian study reported potential savings of USD 593 per patient per year [16]. In healthcare systems operating under fixed or capitated payment schemes, reducing hospitalization-related costs is a key determinant of financial sustainability.

This study aims to estimate the budget impact of adopting HDx compared with HF hemodialysis from the perspective of the Colombian healthcare system, using real-world evidence. It builds upon the COREXH-E study (Colombian Registry of Expanded Hemodialysis–Extension), [17] a retrospective multicenter analysis including 1,098 patients, in which propensity score matching was used to compare hospitalization outcomes between treatment groups.

The COREXH-E study reported a lower annual incidence rate of all-cause hospitalizations among patients treated with MCO membranes (IR = 0.93; 95% CI, 0.82–1.03) compared with HF dialyzers (IR = 1.13; 95% CI, 0.96–1.30), corresponding to a significant incident rate ratio of 0.82 (95% CI, 0.68 to 0.99; *P* = 0.04) [17]. However, given the observational nature of the underlying data, this association should not be interpreted as causal but rather as an estimate of the potential impact of HDx adoption under real-world conditions within the Colombian system. Although propensity score matching reduces imbalance in observed covariates, residual confounding due to unmeasured variables may persist. To address this limitation, the dataset was extended to include an additional pre-intervention year, and a difference-in-differences approach was applied. This method evaluates the impact of MCO membrane use under the assumption of parallel pre-intervention trends, thereby strengthening causal inference [18].

The Colombian healthcare system comprises insurance companies that receive a fixed annual capitation payment for each enrolled individual and independently negotiate the prices of medical procedures with healthcare service providers. While approximately 99% of the population is insured, only around 45% contribute financially to the system through payroll-based contributions, with the remainder is subsidized by the government. Coverage does not extend to all possible health services or every consequence of health deterioration, but only to explicitly non-excluded direct and indirect medical costs. Hemodialysis treatment, hospitalizations, and related procedures and medications are among the services covered by the healthcare system, both in the contributory and subsidized regimes equally [19].

Hemodialysis services in Colombia are reimbursed under bundled payment schemes in which the dialyzer and all treatment-related consumables are included into a single monthly tariff. Similar reimbursement structures are widely implemented across healthcare systems globally. In addition, national regulations prohibit dialyzer reuse; requiring single-use dialyzers for each session.

## Methods

### Study design and overview

**Fig. 1:**
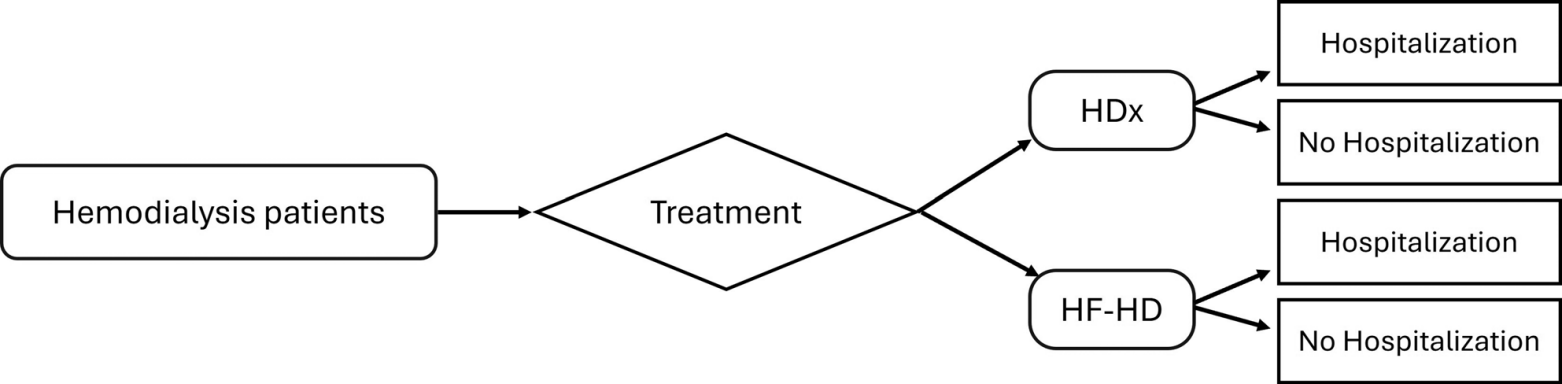
Decision Tree model structure used in the budget impact analysis. The model compares expanded hemodialysis (HDx) with high-flux hemodialysis (HF-HD). Differences in total costs are driven by variation in hospitalization probabilities, while hemodialysis costs remain constant under the reimbursement scheme

A budget impact analysis (BIA) was developed in accordance with the guidelines of the International Society for Pharmacoeconomics and Outcomes Research (ISPOR) [20] to estimate the financial consequences of adopting HDx compared with HF-HD. Under the bundled reimbursement structure, dialysis costs were assumed to be equivalent between treatment alternatives; therefore, differences in total costs were driven by variations in hospitalization rates. The BIA was implemented using a structured Microsoft Excel-based model to estimate changes in total healthcare expenditures under different HDx uptake scenarios. A decision tree model used to estimate the effect of expanded hemodialysis is presented in Fig. 1.

### Perspective and time horizon

The analysis was conducted from the perspective of the Colombian healthcare system, represented by third-party payers (insurance companies). A one-year time horizon was adopted, reflecting the availability of real-world clinical data and aligning with standard practice for budget impact analysis intended to inform short-term financial planning.

### Population and uptake scenarios

The target population comprised adult patients undergoing hemodialysis in Colombia. Data on individuals diagnosed with chronic renal failure in Colombia were obtained from the High-Cost Account records. The average hospitalization rate for this population in Colombia was obtained from the budget sufficiency database of the Colombian Ministry of Health. Records from this dataset were aggregated using all ICD-10 codes relevant to the diagnosis of chronic renal failure. Finally, hospitalization information for each patient was classified according to the reported number of hospital days.

Consistent with the guidelines established by the agency responsible for reviewing budget impact studies in Colombia, we conducted estimations using 5%, 10%, 20%, and 50% as the market use of HDx. These scenarios were selected to reflect the plausible adoption patterns in routine clinical practice and to inform decision-making under varying implementation levels.

### Clinical inputs and real-world evidence

The COREXH-E cohort included 1098 patients treated with hemodialysis, of whom 564 were treated with expanded hemodialysis (HDx) and 534 with high-flux membrane hemodialysis (HF-HD) [17]. The mean age was 60 years, 62% were men, 60% had been on dialysis for more than 3 years, 42% had a history of diabetes mellitus and 19% had cardiovascular disease. See Table 1 for additional details. The primary clinical input for the BIA was the difference in all-cause hospitalization rates between treatment groups.

**Table 1:** Demographic and clinical characteristics of the hemodialysis population

Characteristics	Full sample	HDx	HF-HD	*p*-value
	*n* = 1098	*n* = 564	*n* = 534	
Age, mean (SD) years	60.6 (14.9)	60.8 (15.0)	60.4 (14.9)	0.66
Sex, n (%):				
Male	684 (62.3)	336 (59.6)	348 (65.2)	0.06
Female	414 (37.7)	228 (40.4)	186 (34.8)	
Race, n (%):				
African American	83 (7.6)	28 (5.0)	55 (10.3)	< 0.01
Dialysis vintage, n (%):				
< 1 year	159 (14.5)	70 (12.4)	89 (16.7)	0.13
1 to 3 years	279 (25.4)	148 (26.2)	131 (24.5)	
> 3 years	660 (60.1)	346 (61.4)	314 (58.8)	
Diabetes history, n (%)	460 (41.9)	246 (43.6)	214 (40.1)	0.23
Cardiovascular disease history, n (%)	207 (18.8)	121 (21.5)	86 (16.1)	0.02
Karnofsky scale, mean (SD)	77.1 (14.9)	78.5 (13.8)	75.6 (15.9)	0.01
Body mass index, mean (SD)	24.5 (4.3)	24.9 (4.4)	24.0 (4.1)	< 0.01
Urine output, n (%):				
< 150 ml/day	804 (73.2)	412 (73.1)	392 (73.4)	0.89
>= 150 ml/day	294 (26.8)	152 (26.9)	142 (26.6)	
Albumin, mean (SD) g/dL	4.0 (0.4)	4.0 (0.3)	4.0 (0.5)	< 0.01
Hemoglobin, mean (SD) g/dL	11.7 (1.8)	11.9 (1.7)	11.6 (1.9)	< 0.01
Potassium, mean (SD) mEq/L	5.2 (0.8)	5.2 (0.8)	5.2 (0.8)	0.19
Phosphorus, mean (SD) mg/dL	4.6 (1.5)	4.6 (1.4)	4.6 (1.5)	0.44
Kt/V single pool, mean (SD)	1.6 (0.4)	1.7 (0.4)	1.6 (0.3)	< 0.01
Dialyzer membrane, n (%):				
Revaclear	525 (47.8)	0	525 (98.3)	< 0.01
Polyflux	5 (0.5)	0	5 (0.9)	
Xenium	4 (0.4)	0	4 (0.8)	
Theranova 400	557 (50.7)	557 (98.8)	0	
Theranova 500	7 (0.6)	7 (1.2)	0	
Vascular access, n (%)				
Catheter	195 (17.8)	77 (13.7)	118 (22.1)	< 0.01
Arteriovenous fistula	903 (82.2)	487 (86.4)	416 (77.9)	
QD, mean (SD) ml/min	484 (55.3)	488 (61.5)	480 (47.7)	0.02
QB, mean (SD) ml/min	340 (49.3)	349 (52.1)	330 (43.9)	< 0.01
Follow-up, mean (SD) months	19.1 (8.1)	20.0 (7.4)	18.3 (8.6)	< 0.01

HF=high flux membrane; HDx= Expanded Hemodialysis; QD = Dialysate flow rate; QB = Blood flow rate; SD = standard deviation; Categorical variables were compared with Pearson’s χ2 test and continuous variables were compared with Student’s t-test

Given that treatment groups were not established through random allocation, the COREXH-E dataset was extended to include one year of pre-intervention data to mitigate potential residual confounding. HDx adoption across centers occurred over a relatively short implementation period of approximately three months, reflecting routine clinical, and was therefore treated as quasi-simultaneous. A difference-in-differences (DiD) framework was then applied to compare changes in hospitalization rates over time between patients exposed to HDx and those remaining on HF-HD, under the assumption of parallel trends in the absence of treatment. This assumption was evaluated using an Ordinary Least Square (OLS) model. The dataset was structured as a patient-period panel, with two time periods (pre and post-intervention). This approach allows control for potential unobserved factors, provided that the difference in the outcome variable between the treatment and control groups, which is attributable to unobserved elements remains constant prior to the onset of treatment.

The model was specified as $\:{H}_{it}$represents the hospitalization rate (events per patient-year) for patient i at time t, $\:{Post}_{t}$ is an indicator for the post-intervention period, and $\:{HDx}_{i}$ identifies patients in the treatment group. The interaction term $\:{Post}_{t}$*$\:{HDx}_{i}$ corresponds to the DiD estimator. $\:{X}_{it}\:$is a vector of patient-level clinical and demographic characteristics, $\:{\mu\:}_{c}$ represents healthcare center fixed effects, and $\:{\epsilon}_{it}\:$is an error term. Standard errors were clustered at the healthcare center level to account for within-center correlation. Detailed econometric specifications are provided in the Supplementary Material.

$$\eqalign{ {H_{it}} = & {\beta _0} + {\beta _1}Pos{t_t} + {\beta _2}HD{x_i} \cr & + {\beta _3}(Pos{t_t}*HD{x_i}) + {X_{it}} \cr & + {\mu _C} + { \in _{it}} \cr}$$

To assess the robustness of the results, two tests were conducted. The first involved removing the treatment group indicator from the model and verifying, through comparison of the adjusted R-squared statistic, that the resulting model did not better explain the variation than the model including the variable. This would indicate that accounting for the type of membrane is indeed relevant for explaining hospitalizations. In the second test, aimed at providing evidence that the estimated effect in the original model was not due to chance, the treatment group indicator was replaced with a randomly generated placebo variable. The coefficient on this placebo variable was expected to be statistically non-significant, as assessed using a two-tailed test at the 95% confidence level.

### Cost inputs

Hospitalization costs were obtained from the 2019 budget sufficiency database of the Colombian Ministry of Health and included all-cause inpatient care among patients with chronic kidney disease. Records were aggregated using ICD-10 codes corresponding to the diagnosis of chronic renal failure. During data validation, a subset of hospitalization records with unusually low costs was identified. These values ​​were considered likely to correspond to emergency room visits or priority care, rather than full hospital admissions. To improve the representativeness of hospitalization costs, records with costs below 93 USD were excluded. This threshold was defined through a structured consensus process involving clinical and administrative experts from various institutions. Costs were estimated based on observed resource use and length of stay.

Hemodialysis treatment costs were modeled using a bundled payment approach, consistent with current reimbursement practices in Colombia. A uniform monthly dialysis cost was assumed for both HDx and HF-HD, as reimbursement tariffs do not differentiate by dialyzer type. Consequently, differences in total expenditures between treatment strategies were driven exclusively by differences in hospitalization-related costs.

In the base-case analysis, the cost of the dialyzer was not modeled separately, as it is included within the bundled dialysis tariff under the current Colombian reimbursement system. Therefore, any incremental cost associated with the MCO dialyzer is not reflected from the payer perspective. In an additional scenario analysis, an incremental cost for the HDx was incorporated to simulate a reimbursement setting in which such costs are transferred to the payer.

All costs were expressed in 2025 US dollars. Costs originally reported in 2019 Colombian pesos were adjusted to 2025 Colombian pesos by applying the cumulative inflation rate for the corresponding period, as reported by the Colombian National Administrative Department of Statistics (DANE), and converted using exchange rate of 4,304.57 Colombian pesos per US dollar, reported by the Central Bank of Colombia.

Because the intervention evaluated in this study consists solely of replacing the dialyzer membrane within the same hemodialysis procedure, no additional infrastructure, staffing, or water treatment requirements are generated. Dialysis sessions, dialysis monitors, consumables, and personnel requirements remain unchanged. Therefore, dialysis treatment costs were assumed to be identical between HDx and high-flux hemodialysis under the bundled reimbursement scheme used in the Colombian healthcare system. Consequently, the economic analysis focused on downstream cost differences associated with hospitalization rates.

### Budget impact estimation

The budget impact ($\:\frac{\partial\:G}{\partial\:HDx}$) was calculated as the difference in total annual healthcare costs between a reference scenario in which all patients received HF-HD and alternative scenarios in which a proportion of patients received HDx ($\:\alpha\:$).

$$\eqalign{ & {{\partial \>G} \over {\partial \>HDx}} = \left( {{{\hat G}_{HDx}} - {{\hat G}_{HF - HD}}} \right) \cr & *PatientsInhemodialysis*\alpha ,\alpha \epsilon \left[ {{\rm{0,1}}} \right] \cr} $$

For each uptake scenario, the per-patient difference in annual hospitalization-related costs was multiplied by the projected number of patients transitioning from HF-HD to HDx at the national level. Hemodiafiltration was not included as a comparator in the present analysis because it represents around 4% of hemodialysis patients in Colombia and does not receive differential reimbursement under the current bundled payment system.

### Sensitivity analyses

Deterministic one-way sensitivity analyses were conducted by independently varying hospitalization costs and hospitalization rate differences while holding other parameters constant. Hospitalization cost ranges were defined using the 10th (lower) and 90th (upper) percentiles from the national sufficiency database. Uncertainty in hospitalization rate differences was based on the 95% confidence interval reported in the COREXH-E study.

Additionally, a scenario analysis was conducted incorporating a higher dialyzer cost for HDx. This analysis represents a hypothetical situation in which incremental costs could be transferred to the payer and estimates the maximum proportional increase in the price of MCO membranes that would still allow for potential cost savings associated with their use.

A probabilistic sensitivity analysis (PSA) was performed using 10,000 simulations. In each iteration, hospitalization costs were sampled using a nonparametric bootstrap approach based on observed national administrative data, thereby preserving the empirical distribution of costs. Difference in hospitalization rates between HDx and HF-HD were generated using the inverse probability function with the available confidence interval and assuming a normal distribution.

## Results

### Difference-in-differences analysis

The hospitalization rate per patient-year was lower among patients treated with HDx 1.01 events compared with those receiving HF-HD 1.33 events per patient-year, this estimated reduction in hospitalization frequency constituted the primary clinical parameter driving the budget impact model. The difference-in-differences (DiD) model demonstrated that HDx use was associated with a statistically significant 0.33 reduction in hospitalizations per patient-year compared to high-flux hemodialysis (95% CI: 0.04 to 0.61; *p* = 0.001). This effect size is consistent with previous COREXH-E study findings and supports budget impact estimates.

Assessment of the parallel trends assumption using pre-intervention data showed no statistically significant interaction between treatment groups and time prior to HDx implementation (*p* = 0.188). Pre-intervention hospitalization rates for the treatment and comparison groups exhibit broadly similar trajectories. However, given the limited number of pre-intervention time points, the results suggest an absence of large divergences between the groups before the intervention rather than providing definitive evidence of strictly parallel trends, see Table 2 and Figure S1. The estimated reduction in hospitalization frequency constituted the clinical parameter driving the subsequent budget impact calculations.

**Table 2:** Difference-in-Differences (DiD) regression results for hospitalization rates

Variable	Coefficient	*P*-value	95% CI	
**Parallel trends assumption test**				
HDx * Time	0.046	0.188	-0.022	0.116
**DiD model**				
HDx	-0.328	0.001	-0.612	-0.043
**DiD model with false treatment variable**				
False HDx	0.026	0.855	-0.260	0.313

HDx: Expanded hemodialysis; DiD: Difference-in-Difference

### Budget impact analysis

According to the Colombian Ministry of Health’s budget sufficiency database, which aggregates hospitalization records using ICD-10 codes for chronic renal failure, the national all-cause hospitalization rate among patients receiving hemodialysis was estimated at up to 3.8 events per patient-year across the full insured population (*n* = 25,564). This higher rate reflects comprehensive administrative capture of hospitalization events at the national level and differs from previously reported rates in selected cohorts (1.01 to 1.33 events per patient-year). The mean cost per hospitalization was estimated at USD 1155,8 (2025 values), with a mean length of stay of 15.2 days. Across all modeled uptake scenarios, HDx adoption resulted in net cost savings for the Colombian healthcare system (Table 3 and Table 4).

**Table 3:** Main assumptions used in the budget impact analysis. This table summarizes the key epidemiological, clinical, and economic parameters incorporated into the model. Estimates include the national hemodialysis population, modality-specific annual and monthly costs, hospitalization rates derived from both the DiD analysis and the observed rates in the HD population, the unit cost of hospitalization events, and the one-year analytical time horizon. Market-share scenarios for HDx adoption (5%, 10%, 20%, and 50%) were evaluated to quantify the budgetary implications of incremental uptake under a bundled reimbursement environment

Main assumptions	
Population on hemodialysis in Colombia	25,564
Monthly HF-HD cost per patient	USD 611.4
Monthly cost of HDx per patient	USD 611.4
Annual cost of HF-HD per patient	USD 7,337.3
Annual cost of HDx per patient	USD 7,337.3
HDx hospitalization rate (DiD analysis)	1.01
HF-HD hospitalization rate (DiD analysis)	1.33
HDx hospitalization rate (HD population)	2.87
HF-HD hospitalization rate (HD population)	3.78
Cost per hospitalization event	USD 1,155.8
Time horizon (years)	1
**HDx market share scenarios**	
Share 5%	
Share 10%	
Share 20%	

Share 50%

**Table 4:** Projected annual budget impact of HDx adoption in Colombia

Base case: same package costs, hospitalization rates HDx 1.01 versus HF-HD 1.33 event per patient-year							
Share HDx	Share HF-HD	HDx patients	HF-HD patients	HDx hospitalization events	HF-HD hospitalization events	Total annual cost	Savings
5%	95%	1,278	24,286	1291	32,300	USD 226,396,323.0	USD 472,756.4
10%	90%	2,556	23,008	2582	30,600	USD 225,923,566.6	USD 945,512.8
20%	80%	5,113	20,451	5164	27,200	USD 224,978,053.8	USD 1,891,025.6
50%	50%	12,782	12,782	12,910	17,000	USD 222,141,515.4	USD 4,727,564.0

**Alternative scenario: same package costs**,** hospitalization rates for the HDx population 2.87 versus 3.78 events per patient-year**							
**Share HDx**	**Share HF-HD**	**HDx patients**	**HF-HD patients**	**HDx hospitalization events**	**HF-HD hospitalization events**	**Total annual cost**	**Savings**
5%	95%	1,278	24,286	3668	91,800	USD 297,915,502.1	USD 1,344,401.0
10%	90%	2,556	23,008	7337	86,969	USD 296,571,101.1	USD 2,688,802.0
20%	80%	5,113	20,451	14,674	77,306	USD 293,882,299.1	USD 5,377,604.1
50%	50%	12,782	12,782	36,684	48,316	USD 285,815,893.0	USD 13,444,010.1

HDx: Expanded Hemodialysis; HF-HD: High Flux Hemodialysis; Negative values indicate savings, i.e., a reduction in the total cost

of the Colombian health system through the implementation of HDx; An exchange rate of 4,304.57 Colombian pesos per USD was used for the calculation. The costs were obtained from the Social Protection Information System (SISPRO) database and the sufficiency records of the Per Capita Payment Unit (UPC)

Under hospitalization rates estimated through the DiD approach in the COREXH-E dataset, projected annual savings for the Colombian hemodialysis population ranged from USD 472,756 to USD 4,727,564, corresponding to HDx uptake rates of 5% and 50%, respectively. The magnitude of savings increased proportionally with HDx uptake, reflecting a linear relationship between adoption and reduction in hospitalization-related costs. In scenarios reflecting higher hospitalization rates observed in the general Colombian hemodialysis population, annual savings ranged from USD 1,344,401 to USD 13,444,010 across the same uptake scenarios. These results indicate that the magnitude of economic benefit associated with HDx adoption is sensitive to baseline hospitalization frequency and may be greater in higher-risk populations.

In all modeled scenarios, HDx implementation was associated with reduced total healthcare expenditures relative to continued exclusive use of high-flux hemodialysis (Figure S2).

### Sensitivity analysis

**Fig. 2:**
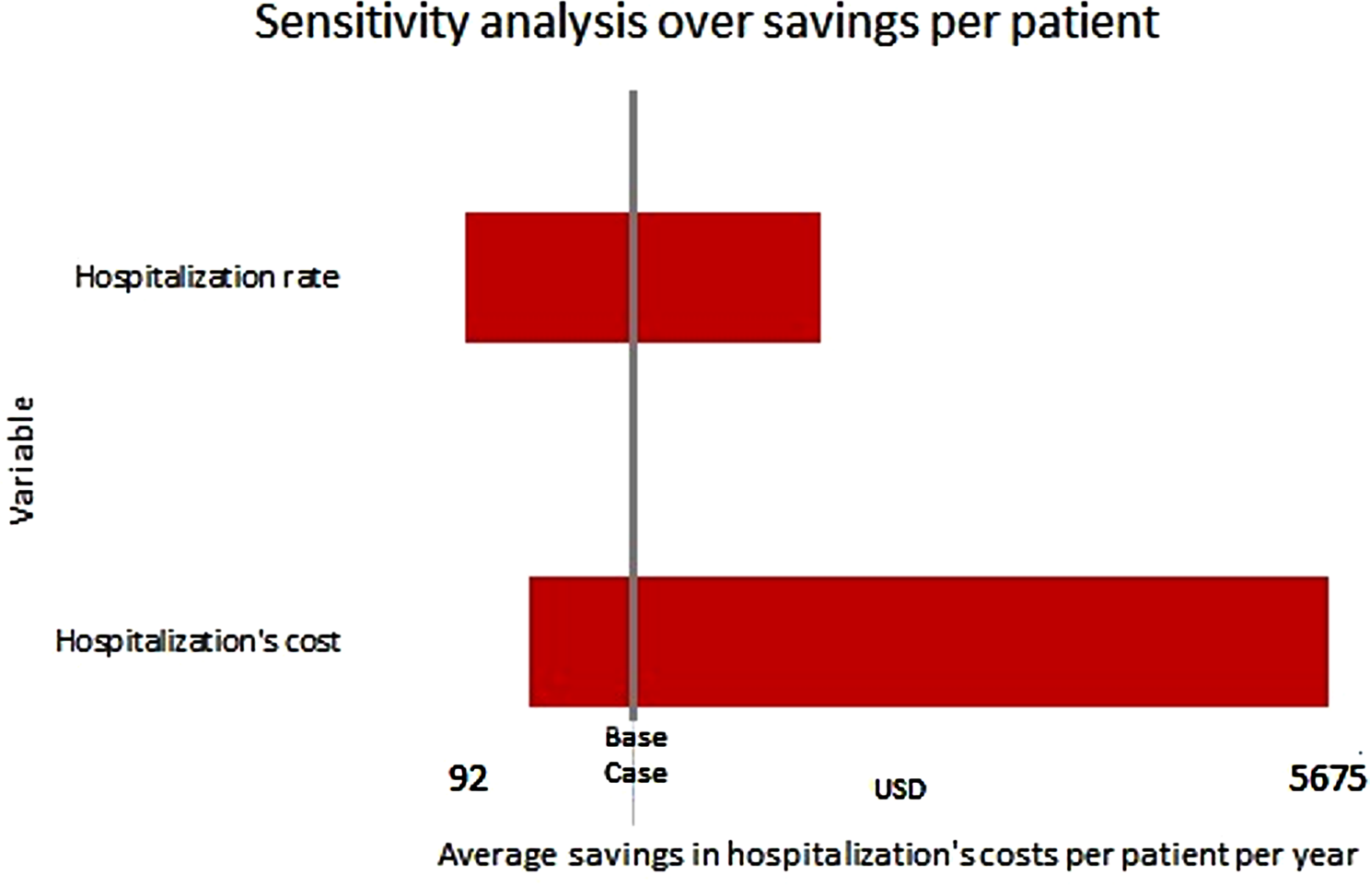
Deterministic sensitivity analysis. The figure shows the impact of variations in hospitalization rates and hospitalization costs on incremental annual cost per patient. The vertical dashed line represents the base-case estimate

Deterministic one-way sensitivity analyses showed that variation in hospitalization costs had the greatest influence on projected savings, with potential per-patient savings varying by up to USD 5675 annually (Fig. 2**)**. Variation in the estimated reduction in hospitalization rates within the confidence interval reported by the DID model, had a comparatively smaller effect on total budget impact.

Given that the annual cost of care was USD 7,337 of which 30% corresponds to the dialyzer, the estimated annual savings amount per patient was USD 381. Under this scenario, the price of the dialyzer would need to increase by approximately 18% for these savings to be fully offset. These estimates reflect the impact derived from hospitalization‑rate reductions obtained through the DiD analysis using data from the COREXH‑E study.

However, when applying the same proportional reduction to the hospitalization rates observed in the population treated with the high‑flux hemodialysis, a patient would be expected to experience 3.78 hospitalizations per year under HF- hemodialysis compared with 2.87 events per year with HDx. Under this alternative scenario, the magnitude of hospitalization reduction is larger, and the dialyzer price could increase by up to 48% before the savings generated by fewer hospitalizations are eliminated.

**Fig. 3:**
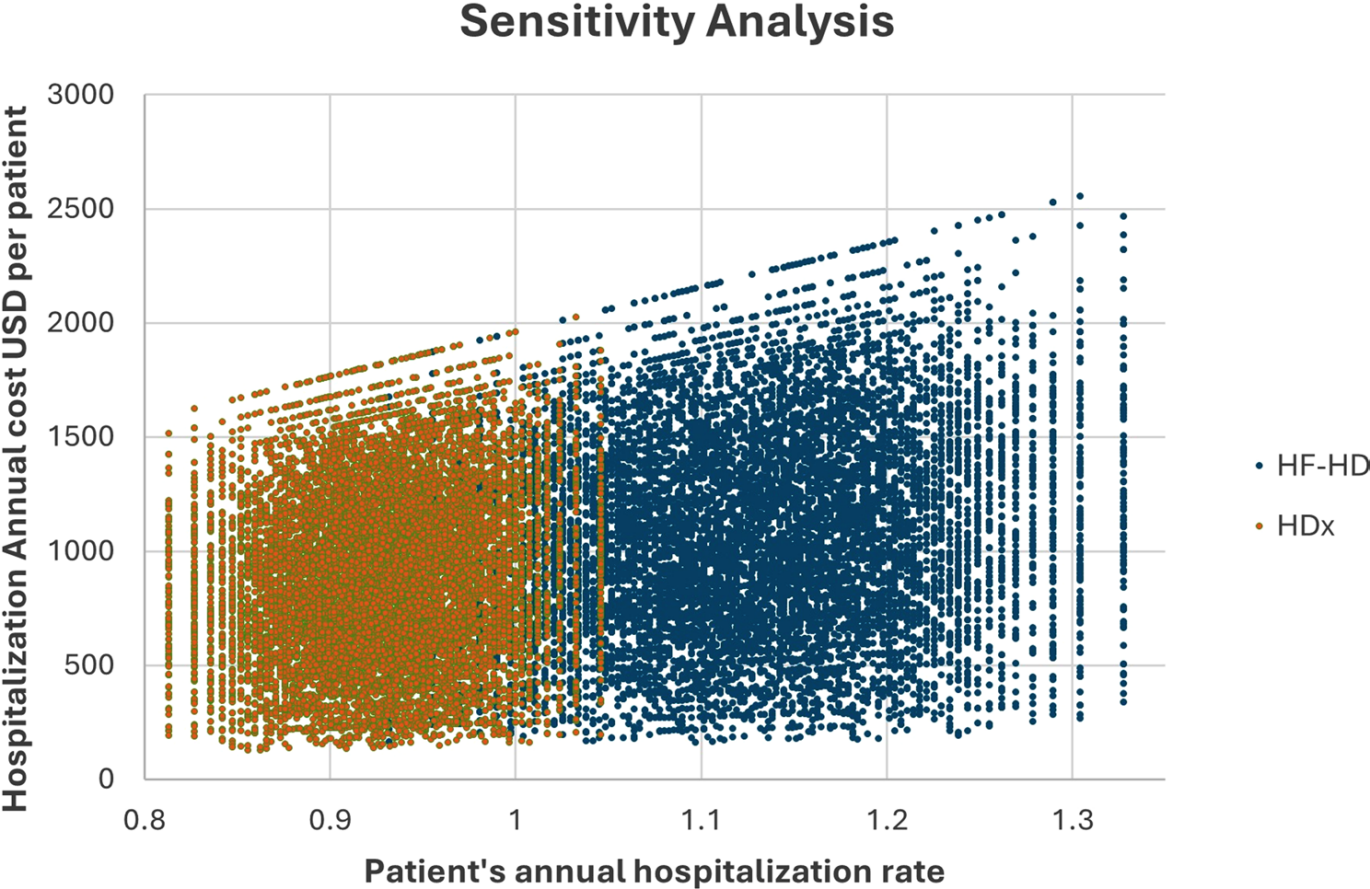
A probabilistic sensitivity analysis. Each dot represents one simulation. The figure illustrates the variability in hospitalization-related costs under both dialysis modalities. Overall, HDx is associated with lower hospitalization rates and reduced annual costs compared to HF-HD

Probabilistic sensitivity analysis based on 10,000 simulations showed that HDx was cost-saving in 97.8% of iterations. These results should be interpreted considering the observational nature of underlying evidence. Although uncertainty in hospitalization costs contributed most to overall variability, the probability of net savings remained consistently high across simulations. Overall, results were robust to parameter uncertainty and consistently favored HDx adoption from a short-term payer perspective, for more details see Fig. 3.

## Discussion

This budget impact analysis indicates that the adoption of expanded hemodialysis (HDx) may generate meaningful short-term cost savings for the Colombian healthcare system, primarily driven by reductions in hospitalization rates. Within a bundled reimbursement framework where dialysis payments are fixed and do not vary by dialyzer type economic gains are not realized through changes in procedure costs, but rather through downstream reductions in healthcare utilization. These findings position HDx as a potentially high-value intervention in systems where reimbursement structures incentivize efficiency at the system level rather than at the level of individual inputs.

Importantly, HDx does not require structural modifications to dialysis delivery. As a membrane substitution within conventional hemodialysis, its implementation does not entail additional infrastructure, staffing, or procedural costs. This characteristic is highly relevant from a health system perspective, as it allows the introduction of a clinically differentiated intervention without disrupting existing care pathways or increasing direct treatment costs under bundled payment schemes.

The observed reduction in hospitalization rates is consistent with prior randomized and observational evidence evaluating medium cut-off membranes, which have been associated with improved clearance of middle molecules and favorable clinical outcomes [16, 21, 22, 23]. While causality cannot be definitively established within the observational framework of this study, the use of a difference-in-differences design strengthens internal validity by accounting for time-invariant confounding. Nevertheless, residual confounding related to patient selection and center-level practices cannot be fully excluded and should be considered when interpreting the magnitude of the estimated effect.

This study extends previous economic evaluations by providing national level estimates within a middle-income country operating under a capitation-based insurance model [19]. Notably, the magnitude of projected savings was highly sensitive to baseline hospitalization rates. When higher hospitalization frequencies derived from national administrative data were incorporated, the estimated economic impact increased substantially. These findings underscore the critical importance of incorporating locally derived epidemiological parameters into budget impact models, as economic outcomes may vary significantly according to population risk profiles and patterns of healthcare utilization.

From a health policy perspective, the findings highlight a structural misalignment between payer and provider incentives. Under the current Colombian reimbursement framework, any incremental cost associated with medium cut-off dialyzers is absorbed by providers, while savings from reduced hospitalizations accrue to payers. This divergence may limit the adoption of HDx despite its potential system level value. Aligning reimbursement mechanisms with demonstrated clinical and economic benefits through strategies such as shared savings models or adjustments to bundled payments may be necessary to facilitate the sustainable uptake of innovations that generate downstream cost reductions.

Although economic evaluations are not inherently generalizable across healthcare systems, the present results may be particularly applicable to settings that employ bundled or capitated reimbursement models. In such contexts, innovations that modify a single component of care without increasing the overall cost of the treatment package can generate net savings at the system level. However, the magnitude and realization of these savings will depend on both the incremental cost of the innovation and its rate of adoption in clinical practice.

Several limitations warrant consideration. First, the analysis was based on observational real-world data, which introduces the potential for residual confounding, including confounding by indication and center-level variability in practice patterns. Although the difference-in-differences approach mitigates bias from unobserved time-invariant factors, it does not fully account for time-varying confounders. In addition, given the limited number of pre-intervention time points, this analysis provides limited evidence regarding the parallel trends assumption. Second, the use of administrative data may be subject to inaccuracies in coding and data completeness; however, this risk was partially mitigated through data cleaning strategies and consistency checks. Third, the analysis was restricted to a one-year time horizon, which limits the capacity to capture longer‑term clinical outcomes.

Finally, the absence of a direct comparison with hemodiafiltration (HDF) should be acknowledged. Although HDF is clinically relevant, its limited use in Colombia and the absence of differential reimbursement reduce its relevance from a payer perspective within the current framework. Future studies evaluating HDx relative to HDF under alternative reimbursement structures may provide additional insight into their comparative value.

## Conclusion

In a national-level analysis conducted within a bundled reimbursement framework, adoption of expanded hemodialysis (HDx) was associated with projected short‑term cost savings, driven primarily by reductions in hospitalization‑related expenditures. Because dialyzer costs are embedded within the fixed payment structure of bundled dialysis reimbursement, these savings are achieved without increasing direct treatment costs from the payer’s perspective. This positions HDx as a high‑value intervention within capitated and bundled payment environments, where efficiency gains translate directly into system‑level savings. Moreover, the findings underscore the importance of reimbursement models that align provider incentives with interventions capable of generating downstream clinical and economic benefits, reinforcing the role of HDx as a strategy that simultaneously enhances patient outcomes and optimizes healthcare spending.

## Supplementary Information

Below is the link to the electronic supplementary material.

Supplementary Material 1

## Data Availability

All data generated or analyzed during this study are included in this article. Further enquiries can be directed to the corresponding author ([mauricio.sanabria@vantive.com](mailto: mauricio.sanabria@vantive.com)).

## Declarations

### Prior presentation

An abridged version of this data was presented as a poster at the International Society of Nephrology congress, Buenos Aires, Argentina, 2024. Kidney International Reports,
