# Supplementary material for: Budget impact of expanded hemodialysis versus high-flux hemodialysis using real-world evidence in Colombia
Source: Cost Eff Resour Alloc. 2026 May 2;24:78. doi: 10.1186/s12962-026-00754-9 (PMC13317427; doi:10.1186/s12962-026-00754-9)

**Supplemental material**

**S1. Econometric Specification of the Difference-in-Differences Model**

Given that treatment assignment was not determined through randomization, a Difference‑in‑Differences (DiD) approach was employed to estimate the effect of HDx on hospitalization outcomes. This quasi‑experimental design allows identification of treatment effects by comparing changes in outcomes over time between a treatment group and a control group, under the assumption that both groups would have followed parallel trends in the absence of the intervention.

The timing of HDx adoption across centers occurred within a relatively short implementation window of approximately three months, reflecting routine clinical rollout rather than a single synchronized start date. Given this limited temporal variation, the adoption pattern can be considered quasi-simultaneous and was appropriately accounted for in the model through the inclusion of time fixed effects

$H_{it}$ = $\beta_{0}$+ ${\beta_{1}Post}_{t}$ + ${\beta_{2}HDx}_{i}$ + $\beta_{3}{(Post}_{t}$*${HDx}_{i}$) + $X_{it}$ + $\mu_{c}$ +$\epsilon_{it}$

Where:

- $H_{it}$ represents the hospitalization rate (events per patient-year) for patient i at time t.

- ${Post}_{t}$ is an indicator for the post-intervention period.

- ${HDx}_{i}$ identifies patients in the treatment group.

- ${Post}_{t}$*${HDx}_{i}$ is the interaction term that represents the DiD estimator.

- $X_{it}$is a vector of patient-level clinical and demographic characteristics.

-$\mu_{c}$ represents healthcare center fixed effects.

-$\epsilon_{it}$is an error term.

**S2. Assessment of the Parallel Trends Assumption**

To evaluate the plausibility of the parallel trends assumption, pre-intervention hospitalization trends were examined using the following specification:

$H_{it}$ = $\beta_{0}$+ ${\beta_{1}Post}_{t}$ + ${\beta_{2}HDx}_{i}$ + $\beta_{3}{(Post}_{t}$*${HDx}_{i}$) + $\epsilon_{it}$

Where:

- $H_{it}$ represents the hospitalization rate (events per patient-year) for patient i at time t.

- ${Post}_{t}$ is an indicator for the post-intervention period.

- ${HDx}_{i}$ identifies patients in the treatment group.

- ${Post}_{t}$*${HDx}_{i}$ is the interaction term that represents the DiD estimator.

-$\epsilon_{it}$is an error term.

**S3. Placebo Test**

A placebo (false treatment) model was estimated to evaluate whether the observed treatment effect could be attributed to random variation.

In this specification, a randomly generated treatment indicator replaced the actual HDx variable in the DiD model. The absence of statistical significance in the placebo interaction term supports the robustness of the estimated treatment effect and reduces the likelihood that findings were driven by chance.

**Figure S1. Pre-intervention assessment of the parallel trends assumption.**

Pre-intervention hospitalization rates for the treatment and comparison groups exhibit broadly similar trajectories. However, due to the limited number of pre-intervention time points, these patterns suggest an absence of large divergences between the groups before the intervention rather than providing definitive evidence of strictly parallel trends.

**
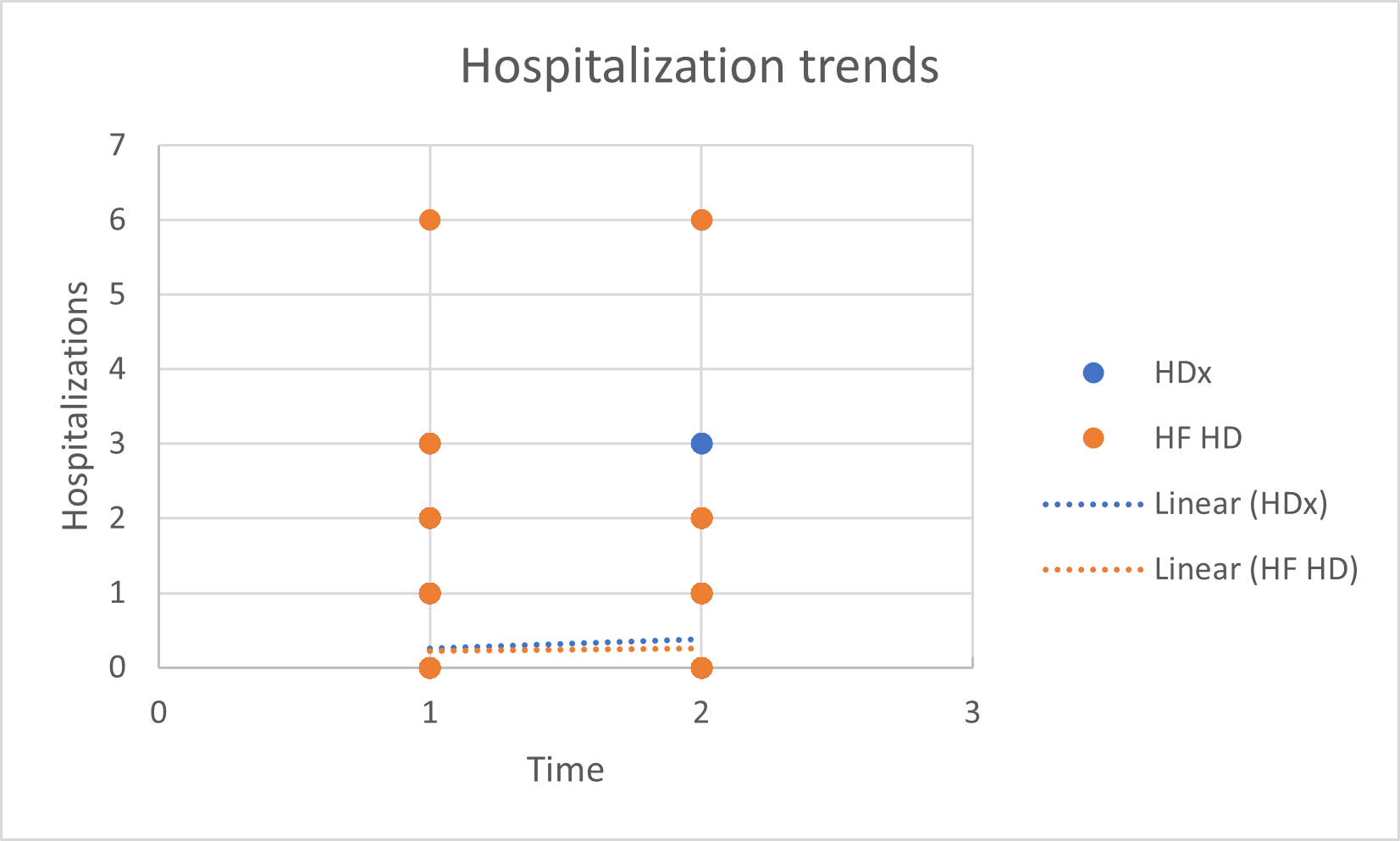
**

**Figure S2. Incremental annual budget impact of HDx adoption across uptake scenarios.** Bars show the incremental annual budget impact associated with HDx adoption at 5%, 10%, 20%, and 50% market uptake compared with a reference scenario of exclusive HF-HD use. Results are shown for the base-case analysis and for a scenario using higher hospitalization rates derived from national administrative data. Negative values indicate cost savings***.***


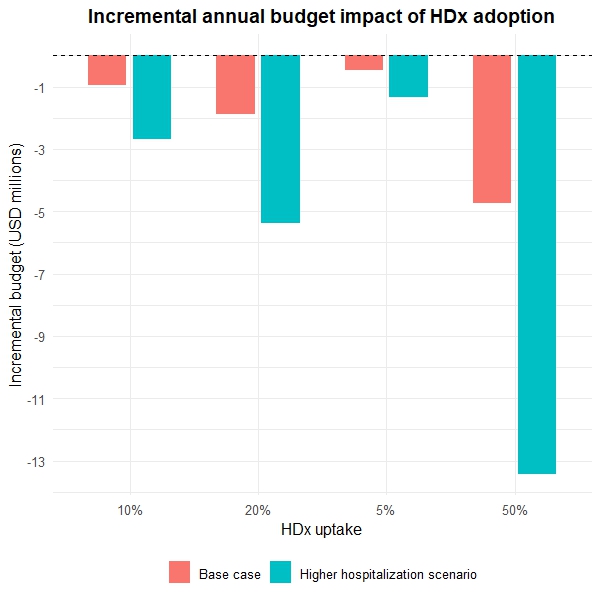

Supplement: Supplementary file 1 — Supplementary Material 1 [file 12962_2026_754_MOESM1_ESM.docx]
